# Incidence, microbiology, and outcomes of endophthalmitis after 111,876 pars plana vitrectomies at a single, tertiary eye care hospital

**DOI:** 10.1371/journal.pone.0191173

**Published:** 2018-01-16

**Authors:** Muna Bhende, Rajiv Raman, Mukesh Jain, Pratik K. Shah, Tarun Sharma, Lingam Gopal, Pramod S. Bhende, Sangeetha Srinivasan, Malathi Jambulingam, Sankara Nethralaya Vitreoretinal Study Group (SNVR-Study Group)

**Affiliations:** 1 Shri Bhagwan Mahavir Vitreoretinal Services, Sankara Nethralaya, Chennai, Tamil Nadu, India; 2 L & T Microbiology Research Centre, Vision Research Foundation, Sankara Nethralaya, Chennai, Tamil Nadu, India; Massachusetts Eye & Ear Infirmary, Harvard Medical School, UNITED STATES

## Abstract

**Purpose:**

To describe the incidence, risk factors, clinical presentation, causative organisms, and outcomes in patients with endophthalmitis following pars plana vitrectomy (20G and minimally invasive vitrectomy surgery (MIVS).

**Methods:**

Of 111,876 vitrectomies (70,585 20-G 41,291 MIVS) performed, 45 cases developed acute-onset, postoperative endophthalmitis.

**Results:**

The rate of culture positive and culture negative endophthalmitis was 0.021% (2.1/10,000 surgeries) and 0.019% (1.9/10,000 surgeries) overall, 0.031% (3.1/10,000 surgeries) and 0.025% (2.5/10,000 surgeries) in 20G, and 0.005% (0.5/10,000 surgeries) and 0.007% (0.7/10,000 surgeries) in the MIVS group respectively. Potential predisposing factors were as follows: diabetes, 46.7%; vitrectomy for vascular retinopathies, 44.4%; and vitrectomy combined with anterior segment surgeries, 35.5%. The culture proven rates were 53.3% overall, 55.0% for 20G and 40.0% for MIVS. The most common organism was *Pseudomonas aeruginosa* for 20G. *Klebsiella and Staphylococcus aureus* were isolated in the two culture positive cases in MIVS group. The follow-up period for the patients with endophthalmitis was 586.14 ± 825.15 days. Seven were lost to follow up beyond one week. Of the remaining 38, 13 (34.2%) cases had a favorable visual outcome (i.e., best-corrected visual acuity [BCVA] > 5/200) and 24 (63.2%) had unfavorable visual outcome (BCVA < 5/200). Group with culture test results negative had significantly better outcomes (*P* < 0.05) as compared to those with positive.

**Conclusions:**

MIVS does not increase the risk of endophthalmitis. Outcomes are poor despite appropriate treatment, particularly in cases with culture results positive.

## Introduction

Endophthalmitis after vitreous surgery is an uncommon but a devastating complication.[1] The reported incidence of endophthalmitis after vitreous surgery ranges from 0.05% to 0.02% with 20-G vitrectomy.[2–10] Initial reports showed higher rates of post-minimally invasive vitreoretinal surgery (MIVS) endophthalmitis, whereas recent reports have shown a declining trend.[6,8,10,11]

A recent multicentric study from India reported incidence rates of endophthalmitis after vitrectomy surgery to be 0.052% with culture-positive endophthalmitis being 0.031%.[12] Although worldwide large multicentric studies have been conducted, these are also not without limitations.[8,10,12,13] These retrospective multicentric studies had among centers heterogeneous case selection criteria, had contrasting antibiotic prophylaxis practice patterns, had different infection control protocols, and had different laboratory and treatment protocols which could affect the anatomical and functional outcomes.

The purpose of this study was to describe the 20-year incidence (20 G and MIVS), causative organisms, and visual outcomes associated with endophthalmitis after pars plana vitrectomy from a single, tertiary eye care center in India. Furthermore, to identify the differences in clinical presentation between post-cataract surgery endophthalmitis and post-pars plana vitrectomy endophthalmitis, we compared our data with previous post-cataract surgery endophthalmitis studies after cataract surgery from India. To the best of our knowledge, this is the largest case series of incidence of post-vitrectomy endophthalmitis reported in literature.

## Methods

### Study population

Approval of the Institutional Review Board of Sankara Netharalaya was obtained to analyze the hospital-based data, and the tenets of the Declaration of Helsinki were followed. This was a retrospective review of case records of patients with suspected postoperative infectious endophthalmitis after pars plana vitreous surgery performed at a tertiary eye care institute in India was done. Data were collected from the electronic/paper medical records between January 1995 and December 2015; the charts of all patients who developed endophthalmitis were reviewed. For cross-validation of our data, the endophthalmitis surveillance log of the Department of Microbiology was reviewed. Acute endophthalmitis after vitrectomy was defined as the presence of unusual inflammation in the anterior segment and vitreous cavity within 6 weeks of surgery along with microbiological evidence (staining/culture/PCR) of bacterial and fungal infection. Culture-positive endophthalmitis was considered to be present if microbiological organisms were isolated on culture. If the fungal, aerobic, and anaerobic culture results were found to be all negative, then this case was considered to be culture-negative endophthalmitis. Polymerase chain reaction (PCR) data on the presence or absence of eubacterial, *Propionibacterium acnes*, and fungal genomes were also noted.

Data such as presenting complaints, systemic illness, time of presentation, presenting and final visual acuity, clinical findings, intraoperative procedure, microbial profile of aqueous, vitreous, or any other tissue/material, antimicrobial susceptibility, management including both intravitreal antibiotics and vitrectomy, and follow-up were collected. Informed written consent was obtained to have their medical records used in research.

### Surgical procedure details

Before 2009, all cases underwent 20-G pars plana vitrectomy. After that the practice shifted to MIVS and the number of cases undergoing 20-G vitrectomy declined considerably. Of the 111,876 vitrectomies performed during the study period, 70,585 (63.1%) were 20-G surgeries and 41,291 (36.9%) were MIVS. Before the year 2000 the hospital infection control protocol included pre-operative antibiotics (sulfacetamide, 10.0%) eye drops four times per day for atleast 3 days prior to surgery. Intra-operatively, peri-ocular cleaning was done with 0.5% cetrimide solution (Cettan, Tansi Polish Unit, Chennai). After the year 2000, preoperative antibiotic protocol included instillation of ciprofloxacin (0.3%) or sulfacetamide (10.0%) eyedrops 2 hourly one day before surgery. Pre-operatively 5% povidone-iodine solution was instilled in conjunctival sac and was allowed a contact period of 3 minutes followed by peri-ocular cleaning with 1% povidone iodine solution before draping. The details of indication for surgery, additional procedures performed along with vitrectomy, gauge of vitrectomy instruments, lens status at the end of vitreous surgery, type of tamponade used during surgery, and surgical time were noted in all the cases who developed endophthalmitis.

### Procedures done in presence of endophthalmitis

On clinical suspicion of endophthalmitis, if vitreous surgery was not contemplated in the next 6 hours, an anterior chamber tap was performed and the samples were subjected to gram staining, KOH staining, bacterial and fungal cultures, antibiotic sensitivity, and PCR for *P*. *acnes*, eubacterial and panfungal genomes. If surgery was contemplated, vitreous specimen was collected during vitrectomy and similar microbiological tests were conducted. On the basis of microbiological results, initial antibiotics were given. However, in cases of initial negative results of staining, choice of antibiotics was based on clinical judgment. Scleral or corneal scraping were taken for microbiological evaluation, when indicated.

### Statistical analysis

Statistical analysis was performed using SPSS Statistics for Windows version 21.0. Continuous variables were expressed as mean ± SD and were analyzed using Mann–Whitney *U* test. Categorical variables were analyzed using Chi-Square and Fisher exact test, as applicable. The *P* values of ≤0.05 were considered to be statistically significant.

## Results

**Fig 1:**
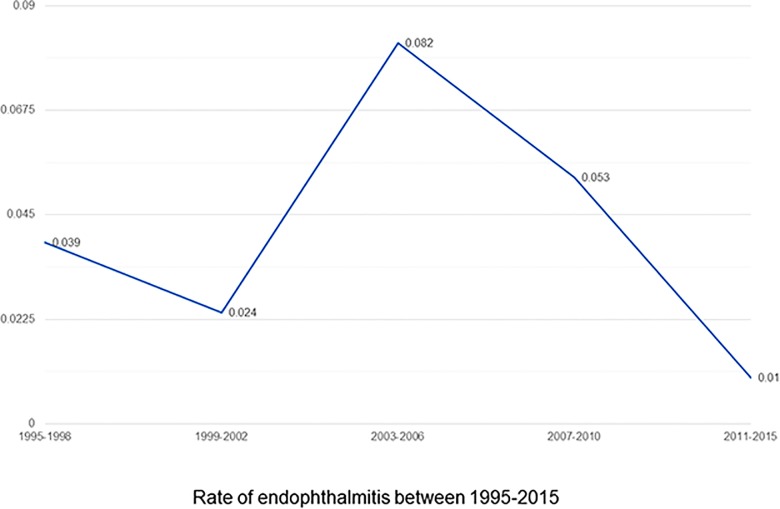
The trend in the rate of endophthalmitis for the study period of 1995–2015.

Table 1 shows the incidence rates of endophthalmitis after pars plana vitrectomy in 20G and MIVS group. The overall incidence of clinically evident and culture-proven endophthalmitis after vitrectomy was 0.040% (4.0 cases per 10,000 surgeries) and 0.021% (2.1 cases per 10,000 surgeries), respectively. The incidence of clinically evident and culture-proven endophthalmitis after 20G vitrectomy was 0.057% (5.7 cases per 10,000 surgeries) and 0.031% (3.1 cases per 10,000 surgeries), and 0.012% (1.2 case per 10,000 surgeries, *P* < 0.05) and 0.005% (0.5 cases per 100,000 surgeries, *P* < 0.05) in the MIVS group, respectively. The rate of culture negative endophthalmitis was 0.019% (1.9/10,000 surgeries), 0.025% (2.5/10,000 surgeries) in 20G and 0.007% (0.7/10,000 surgeries) in the overall, 20G and MIVS group respectively. Fig 1 shows the trend in the rate of post-vitrectomy endophthalmitis for the study period of 1995–2015. Over the last decade the has been there is an indisputable decrease noticed in the rate of endophthalmitis post-vitrectomy; the rate decreasing from 0.082% for the study period of 2003–2006 to 0.01% for the study period of 2011–2015.

**Table 1:** Incidence of post-operative endophthalmitis after vitreous surgery using 20G Vs MIVS vitrectomy.

Type	Overall Group (n = 111876)		20 G Surgeries (n = 70585)		MIVS Surgeries (n = 41291)		p	OR	95% C.I
	Number	Incidence	Number	Incidence	Number	Incidence			
Endophthalmitis	45	0.040%	40	0.057%	5	0.012%	<0.001*	4.68	1.85–11.86
Culture Positive Endophthalmtis	24	0.021%	22	0.031%	2	0.005%	0.004*	6.44	1.51–27.37
Culture Negative Endophthalmtis	21	0.019%	18	0.025%	3	0.007%	0.032*	3.51	1.03–11.92

*p is statistically significant;

C.I: Confidence interval, OR: Odds ratio for endophthalmitis in 20G

Table 2 shows the baseline characteristics and surgical details of the 45 postvitrectomy endophthalmitis patients in the 20-G versus MIVS group. Overall, the group had significant proportion of patients with altered immune status (diabetes, 46.7%; and those on peri-operative oral steroids, 11.1%). One (2.2%) patient had an infective focus (tooth caries). 7 (15.5%) of 45 eyes had intraocular surgery within 1 year prior to vitrectomy (MIVS, 3 eyes; and 20 G, 4 eyes). The most common indications for vitrectomy were vascular retinopathies (44.4%), followed by rhegmatogenous retinal detachment (17.8%). In 16 (35.5%) eyes, vitrectomy was combined with an anterior segment procedure. There were no differences between the two gauges in terms of duration of surgery, number of sclerotomies, use of tamponade, and the lens status at closure. Of the total 111,876 cases, 8 (of 19741 cases) and 37 (of 92135 cases) cases of endophthalmitis occurred before and after introduction of pre-operative povidone iodine drops. Thus, an incidence rate of endophthalmitis before and after the introduction of pre-operative povidone iodine drops were 0.041% and 0.040% respectively (p>0.05).

**Table 2:** Baseline characteristics of patients with endophthalmitis in 20G vs MIVS group.

Baseline characteristics		20 G (n = 40)	MIVS (n = 5)	Overall (n = 45)	p-value
Age	Less than 50 years	18 (45.0%)	4 (80.0%)	22 (48.9%)	0.172
	More than 50 years	22 (55.0%)	1 (20.0%)	23 (51.1%)	
Sex	Male	26 (65.0%)	5 (100%)	31 (68.9%)	0.235
	Female	14 (35.0%)	0 (0.0%)	14 (31.1%)	
Immune predisposition	None	16 (40.0%)	3 (60.0%)	19 (42.2%)	
	Diabetes	19 (47.5%)	2 (40.0%)	21 (46.7%)	0.808
	Steroids (oral)	5 (12.5%)	0 (0.0%)	5 (11.1%)	
Any infective foci	Tooth caries	1 (2.5%)	0 (0.0%)	1 (2.2%)	
Intra-ocular surgery within 1 year prior to vitrectomy	Yes	4 (10.0%)	3 (60.0%)	7 (15.5%)	**0.004**
Indication for PPV	Vitrectomy for Vascular retinopathies	18 (45.0%)	2 (40.0%)	20 (44.4%)	0.832
	Vitrectomy for uveitis	6 (15.0%)	0 (0.0%)	6(13.3%)	
	Vitrectomy for macular pathologies	4 (10.0%)	1 (20.0%)	5 (11.1%)	0.502
	Traumatic vitreous hemorrhage	1 (2.5%)	1 (20.0%)	2 (4.4%)	0.074
	ROP	1 (2.5%)	0 (0.0%)	1 (2.2%)	
	RRD	8 (20.0%)	0 (0.0%)	8 (17.8%)	
	Nucleus drop/ sub-luxated lens	2 (5.0%)	1 (20.0%)	3 (6.7%)	0.205
Procedure performed	Vitrectomy alone	25 (62.5%)	4 (80.0%)	29 (64.4%)	0.641
	Vitrectomy + anterior segment procedure combined	15 (37.5%)	1 (20.0%)	16 (35.5%)	
Duration of Surgery (hrs)	≤2 hrs >2 hrs	21 (52.5%) 19 (47.5%)	4 (80.0%) 1 (20.0%)	25 (55.5%) 20 (44.4%)	0.362
No. of Sclerotomies	3	39 (97.5%)	5 (100%)	44 (97.8%)	1.000
	4	1 (2.5%)	0 (0.0%)	1 (2.2%)	
Tamponade	No tamponade	22 (55.0%)	5 (100%)	27 (60.0%)	0.073
	Tamponade	18 (45.0%)	0 (0.0%)	18 (40.0%)	
Lens status at closure	Phakic	17 (42.5%)	1 (20.0%)	18 (40.0%)	0.634
	Not Phakic	23 (57.5%)	4 (80.0%)	27 (60.0%)	

PPV: Pars plan vitrectomy, Sx: surgery, ROP: retinopathy of prematurity, RRD: Rhegmatogenous retinal detachment, SOI: silicone oil, C3F8: Perfluropropane

We compared our data with historical data on endophthalmitis after cataract surgery to identify the differences of clinical presentation for endophthalmitis after cataract surgery versus vitrectomy.[14] Table 3 shows the difference in clinical presentation between postvitrectomy and postcataract surgery endophthalmitis groups: 73.4% versus 50% patients in the postvitrectomy and postcataract surgery groups, respectively, presented within the first week (*P* = 0.006). Presence of fibrin was a finding in 93.3% patients in the postvitrectomy group compared to 72.5% patients in the postcataract surgery group (*P* = 0.004). Corneal edema and absence of red fundal glow (Grade 5) was more common in the postcataract surgery group compared to the postvitrectomy group (*P* = 0.001). Only 44.4% patients in the postvitrectomy group had normal intraocular pressure (IOP). Low and high IOP was recorded in 8.9% and 46.6% patients in the postvitrectomy endophthalmitis surgery group. This is in contrast to that reported by Endophthalmitis Vitrectomy Study (EVS), where normal IOP was seen in 80.6% of patients.[15] Postoperative hypotony (defined by IOP < 7 mmHg) was seen in one (20%) patient in the MIVS group. Presence of corneal ulcer and scleral necrosis involving the sclerotomies were findings specific to this study.

**Table 3:** Comparison of clinical profile of post vitrectomy endophthalmitis with historical information on post cataract surgery endophthalmitis.

Feature		Present study Endophthalmitis after Vitreous surgery (n = 45)	Previous studies Endophthalmitis after cataract surgery (n = 124)	p-values
First presentation	Mean (SD) (in days)	8.83 (12.0)	**15.8+24.0**^@^	0.064
	1^st^ week	73.4%	50%^@^	**0.006**
	2^nd^ week and beyond	26.6%	50%	**0.006**
Anterior Segment	Presence of fibrin	93.3%	72.5^@^	**0.004**
	Presence of corneal edema	44.4%	**73.5%**^@^	**0.001**
	Presence of hypopyon	53.3%	50%^@^	0.705
Intra-ocular pressure	Low	8.9%	-	
	Normal	44.4%	-	
	High	46.6%	-	
Vitreous involvement	Vitritis	62.2%	-	
	Vitreous exudate	17.8%	-	
	No view	37.8%	65.5%^@^	**0.001**
Choroidal detachment		2.2%	-	
Corneal ulcer		2.2%	-	
Scleral necrosis		4.4%	-	

^@^Gupta A, Gupta V, Dogra MR, Pandav SS, Ray P, Chakraborty A. Spectrum and clinical profile of post cataract surgery endophthalmitis in North India. Indian journal of ophthalmology. 2003 Jun 1;51(2):139.

**Fig 2:**
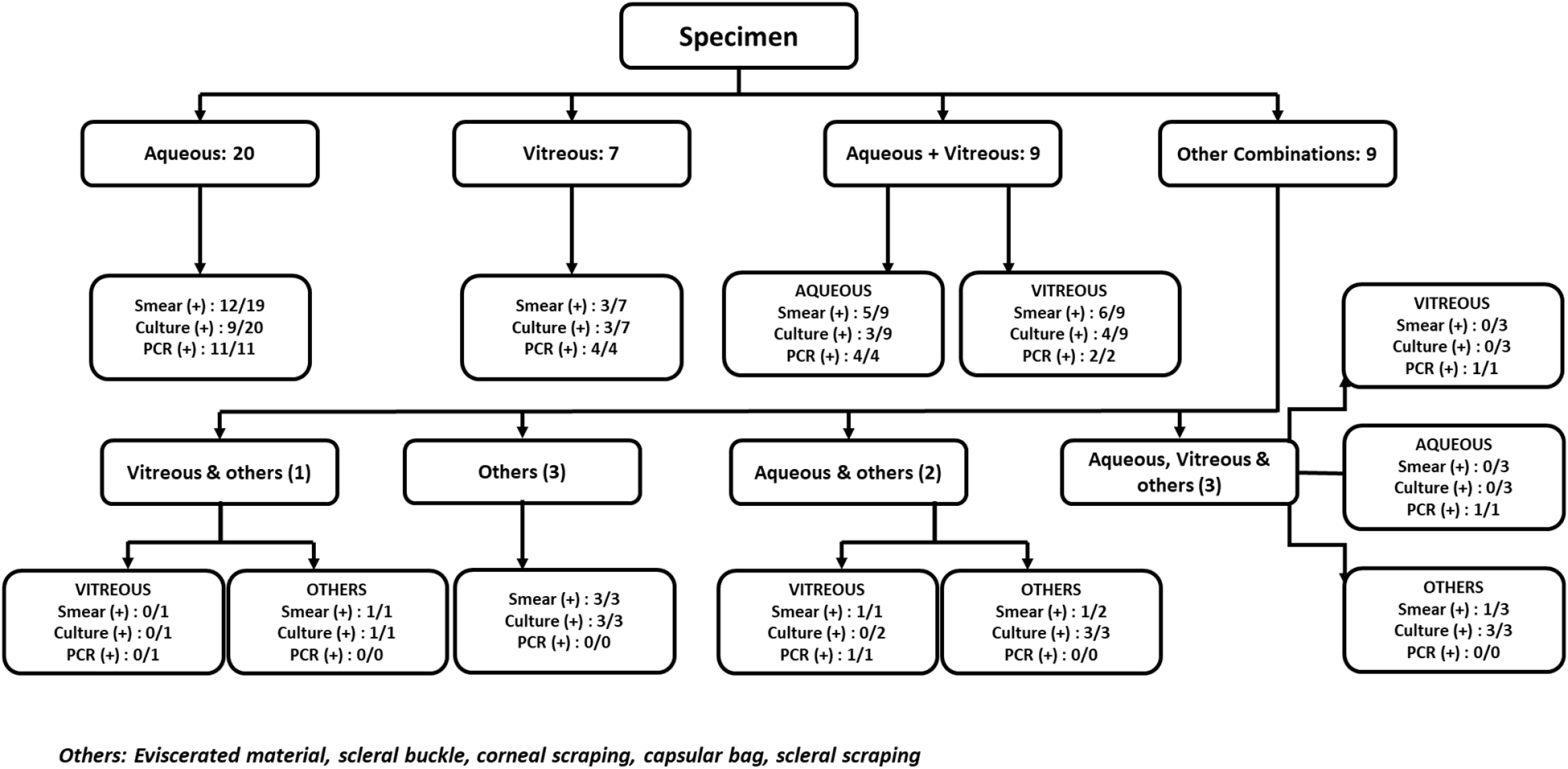
Flow-chart showing the staining, culture and PCR results in various specimen.

Fig 2 shows the various intraocular samples taken and microbiological results obtained. The mean number of samples obtained was 1.4 per patient. Of the total 63 intraocular samples obtained, aqueous, vitreous, and others constituted 33 (52.4%), 20 (44.4%), and 10 (22.2%) samples, respectively. Smear, culture, and PCR positivity in aqueous samples was 56.2% (18/32), 33.3% (11/33), and 100% (17/17), respectively, and in vitreous samples was 45% (9/20), 35% (7/20%), and 87.5% (7/8), respectively. Overall smear, culture, and PCR positivity in both the groups was 54.5% (30/55), 38.6% (22/57), and 96% (24/25), respectively, in our case series.

**Fig 3:**
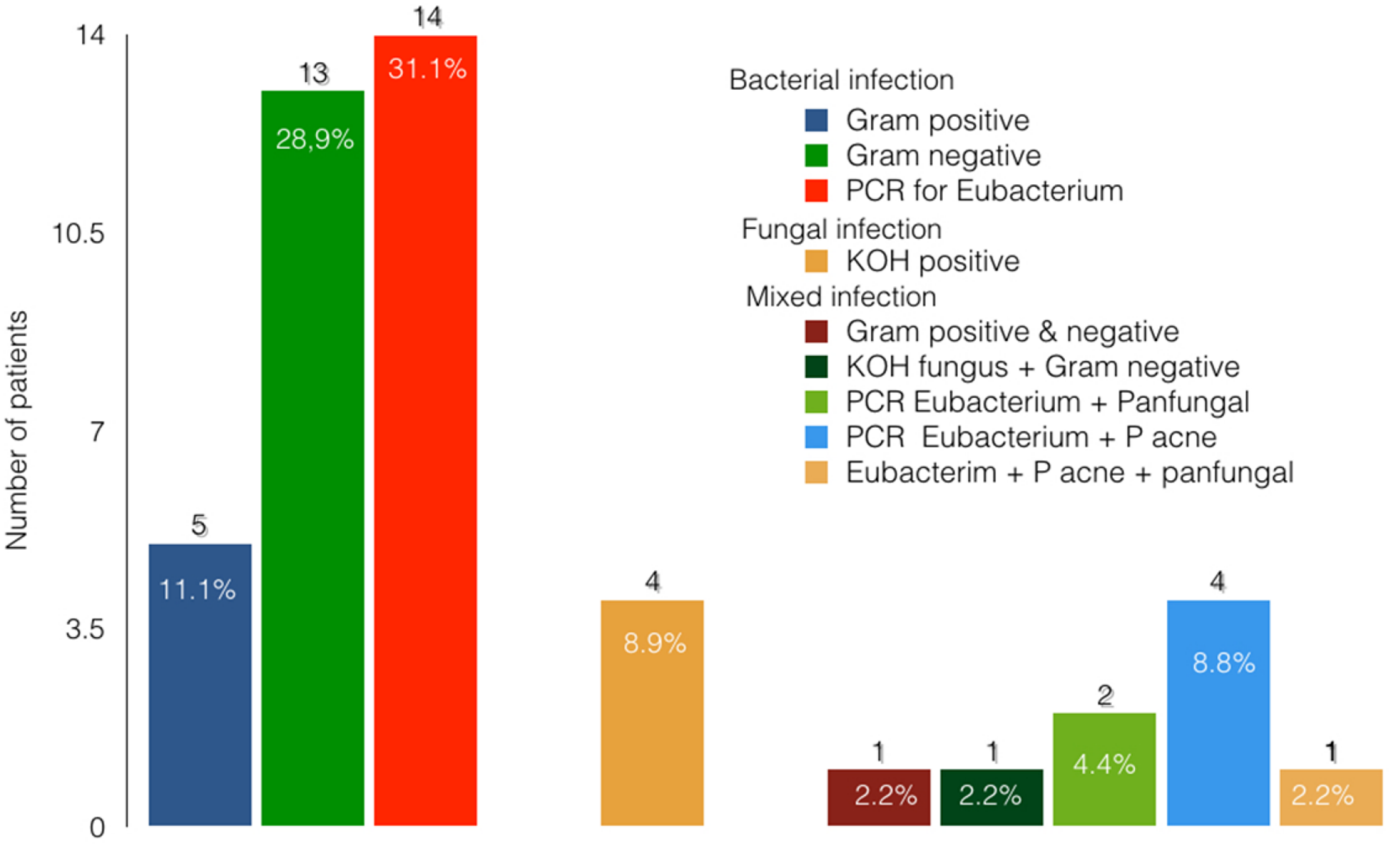
Microbiological profile of ocular specimen in cases with post-operative endophthalmitis.

Fig 3 shows the microbiology findings of 45 postvitrectomy endophthalmitis patients. Culture results of 24 (53.3%) cases were found to be positive in our series. The culture proven rates were 55.0% for 20G and 40.0% for MIVS, respectively. The organisms isolated in the 24 culture-proven cases were, in decreasing frequency,: gram-negative organisms, 12/24 (50.0%); gram-positive organisms, 5/24 (20.8%); fungus, 4/24 (16.7%); *P*. *acnes*, 1/24 (4.2%); combined gram-negative and gram-positive organisms, 1/24 (4.2%); and combined bacterial and fungal, 1/24 (4.2%). The common organisms isolated in our case series were: *Pseudomonas aeruginosa*, 5/24 (20.8%); *Staphylococcus epidermidis*, 2/24 (8.3%); *Staphylococcus aureus*, 2/24 (8.3%); *Aeromonas hydrophila*, 2/24 (8.3%); *Acinetobacter calcoaceticus*, 3/24 (12.5%); and *Aspergillus species*, 5/24 (20.8%). In one case each, *Enterococcus faecalis* (4.2%), *P*. *acnes* (4.2%), *Alcaligenes faecalis* (4.2%), *Corynebacterium* (4.2%), *Klebsiella ozaenae* (4.2%), and *Pseudomonas stutzeri* (4.2%) was isolated.

S1 Table shows the antibiotic sensitivity of the cases with positive cultures. The antibiotic sensitivity of gram-negative organisms was as follows: amikacin, 9/11 (81.8%); ciprofloxacin, 9/11 (81.8%); ceftazidime, 7/9 (77.8%); gentamicin, 9/12 (75.0%); and cefatoxime, 7/11 (63.6%) In gram-positive organisms, the antibiotic sensitivity pattern was as follows: cefatoxime, 4/4 (100%); ciprofloxacin, 4/4 (100%); vancomycin, 3/3 (100%); clindamycin, 3/4 (75%); and ceftazidime, 2/3 (66.7%). Of the 21 cases whose culture results were found to be negative, PCR results were positive in 20 (95.2%) and smear results were positive in only 1 (4.8%) patient (gonococcal bacilli). The results of PCR were as follows: eubacterial only (45%); eubacterial + pan fungal (15%); eubacterial + *P*. *acnes* (30%); and eubacterial + pan fungal + *P*. *acnes* (10%).

Seven eyes were lost to follow up beyond one week and were excluded from further analysis. The follow-up period for the patients with endophthalmitis was 586.14 ± 825.15 days. Table 4 shows the overall visual outcomes in various subgroups. Overall, 13 (34.2%) patients had a favorable visual outcome (i.e., best-corrected visual acuity [BCVA] > 5/200), and 24 (63.2%) had an unfavorable visual outcome (BCVA < 5/200). Overall, 5 (13.1%) and 10 (26.3%) patients had a vision of better than or equal to 6/18 and 6/60, respectively. The globe could be salvaged in 27 (71.0%) cases. The most common cause of poor vision was macular pathology, including scar, epiretinal membrane, and thinning across the three groups. No statistically significant difference was observed in outcome in different subgroups: gram negative versus gram positive; those who had tamponade versus those who did not have tamponade. However, cases with positive culture (particularly gram-negative and fungus-infected) had more unfavorable outcomes (*P* < 0.05). Although no significant difference was observed in the clinical presentation between the groups (*P* > 0.05), patients in the culture-positive group presented relatively early compared to the culture-negative group (75.0% vs. 66.7% at week 1). A higher proportion of patients in the culture-negative group were treated with only intraocular antibiotics (IOAB) compared to those in the culture-positive group (52.4% vs. 25.0%; *P* = 0.120).

**Table 4:** Functional outcome of patients with endophthalmitis in various groups.

	Favorable (BCVA up to 5/200)	Unfavorable (worse than 5/200)	p value	Not applicable (Pediatric patient)	lost to follow-up
Culture positive (n = 24)^#^	4 (16.7%)	16 (66.7%)	0.021**	0 (0.0%)	4 (16.7%)
Culture negative (n = 21)	10 (47.6%)	7 (33.3%)		1 (4.8%)	3 (14.3%)
Gram negative (n = 14)	1 (7.1%)	11 (78.6%)	<0.001**	0 (0.0%)	2 (14.2%)
Gram positive (n = 7)	1 (14.3%)	4 (57.1%)	0.079	0 (0.0%)	2 (28.6%)
Fungal (n = 5)	1 (20.0%)	4 (80.0%)	0.058	0 (0.0%)	0 (0.0%)
No tamponade (n-27)	9 (33.3%)	16 (59.2%)	0.735	0 (0.0%)	2 (7.4%)
Tamponade (n = 18)	5 (27.8%)	7 (38.9%)		1 (5.5%)	5 (27.8%)
Overall (n = 38)*	13 (34.2%)	24 (63.2%)		1 (2.6%)	0*

*- 7 patients lost to follow up were excluded from analysis in the overall group;

**—difference is statistically significant (p<0.05);

^#^: 1 case had gram positive and gram negative bacteria isolated on culture, 1 case had gram negative and fungus isolated on culture

## Discussion

We report the clinical features, microbiological profile, and outcomes in 45 cases of endophthalmitis following 111,876 vitrectomies performed over a period of 20 years. The incidence of clinically evident and culture-proven endophthalmitis after 20G vitrectomy was 5.7 and 3.1 cases per 10,000 surgeries, respectively. Previous studies have shown varied incidences with 20-G vitrectomy: 7 cases per 10,000 surgeries in earlier studies and reducing to 2 cases per 10,000 surgeries reported in the later studies.[2–10]

The incidence of endophthalmitis after MIVS reported in literature in the last decade have shown a declining trend. In a large, multicenter retrospective Pan-American Collaborative Retina Study Group, Wu et al.[10] found an incidence of 0.026% (2.6 cases per 10,000 surgeries) for endophthalmitis after MIVS during 2005–2009. Scott et al.[6] in their multicenter retrospective study found an incidence of 0.048% (4.8 cases per 10,000 surgeries) for endophthalmitis after MIVS during 2007–2008. They also found that the rate of endophthalmitis after MIVS in their study period was marginally lower than that estimated for 2005–2006, suggesting the decreasing trend. Similar indisputable decreasing trend is evident in our study (Fig 1) with the rate decreasing from 0.082% to 0.010% in the last decade. Lower rates observed in our study compared with previous studies are possibly due to the various reasons. As the incidence of endophthalmitis in the MIVS group is calculated for 2009–2014, we ascribe these lower rates to the decreasing trend observed in the past.[6,8,10,11] Also, as our data represent the incidence from a single center, data are possibly less heterogeneous in terms of inclusion criteria, antibiotic prophylaxis, standard infection prevention protocols, and surgical technique except for those that have evolved over time. Moreover, the inclusion criteria of our study is more robust. We included only those cases that had an unusual post-operative inflammation sufficient enough to warrant a microbiological evaluation of ocular fluids and had microbiological evidence (staining positive/culture positive/PCR positive) of the same.

Although we found that the risk of endophthalmitis is significantly lower after MIVS compared to conventional 20-G vitrectomy, caution is warranted in interpreting this finding. As the incidence rates of endophthalmitis after 20-G vitrectomy and MIVS represent different study periods, comparing the two may not reflect the true difference. Since the first introduction of 25-G vitrectomy by Fujii et al.[16] in 2002, the incisional techniques and technology of MIVS have evolved considerably, exemplified by the reported of decreasing incidence of endophthalmitis in the last decade. At best, our results suggest no increased risk of endophthalmitis after MIVS.

The potential predisposing factors identified in our study were as follows: diabetes (46.7%), vitrectomy for vascular retinopathies (44.4%), vitrectomy combined with anterior segment procedures (35.5%), and absence of tamponade (59.5%). History of intraocular surgery was more often seen in patients in the MIVS group. Diabetes, and immune-compromised states, can theoretically increase the risk of endophthalmitis after vitrectomy. Complicated vitreoretinal surgeries requiring multiple exchange of instruments, longer operative time, increased incidence of cataract requiring combined anterior segment surgeries, and poor wound healing can increase the risk of endophthalmitis. Similar associations have been noted in other studies.[1–5,7,9]^1–5,7,9^

The differential surface tension of a tamponading agent compared to balanced salt solution helps to seal the sclerotomy wounds, minimizing hypotony and associated infections.[17–19] Although this study has not been designed to show the association of use of tamponade and endophthalmitis, we found absence of tamponading agent in all cases with endophthalmitis (*n* = 5) who underwent MIVS where sclerotomies were self sealing. Park et al.[11] in their series found that vitrectomy for retinal detachment was associated with reduced risk of infection. They suggested that as in these cases the use of a tamponading agent probably results in better wound integrity.

Because most patients have varying degree of pain, redness, chemosis, lid edema, and anterior segment inflammation, recognizing endophthalmitis early is sometimes challenging. We found a different presentation of endophthalmitis after postvitrectomy compared to that after cataract surgery in terms of fibrin, corneal edema, and IOP, suggesting a differential response of eyes in these two conditions.[14–15] We found that a significant proportion of postvitrectomy endophthalmitis patients (73.4%) presented within 1 week of surgery, which is in accordance with the results of the previous studies. This implies that after vitrectomy, patients must be reviewed in the first week to allow early detection of this dreaded complication. With the presence of fibrin (present in 93.3% patients) and abnormal IOP (present in 55.6% patients, especially high IOP), clinician should suspect endophthalmitis as an important differential diagnosis. One additional finding in patients of postvitrectomy endophthalmitis was the presence of corneal ulcer and scleral necrosis in the area of sclerotomy. This indicates that meticulous examination of the sclerotomy site is important in the post-operative period.

We found 53.3% culture positivity in our case series, which is in accordance with previously reported rates between 44.4% and 66.7%.[6,8,9,11,20] Park et al.[20] suggested the importance of aqueous samples in isolating the microorganisms in endophthalmitis after vitrectomy. We found that aqueous was the most common intraocular sample obtained (52.4%) with a culture-positive yield similar to that of vitreous samples (33.3% vs. 35.0%), further validating its use.

An important distinctive feature of our study is the use of PCR for microbial analysis. Chiquet et al.[21] found culture positivity in only 32% aqueous samples as compared to 61% on PCR in their postcataract surgery endophthalmitis case series study. Using culture and PCR combination, diagnosis can be made in 71% cases. Similarly, Therese et al.[22] showed that by inclusion of PCR, detection of microorganisms increased from 46.5% to 75.8% cases. Likewise, Anand et al.[23] showed a high sensitivity for detection of fungus using PCR in endophthalmitis.

Reports from Western countries have shown coagulase negative *Staphylococcus* as the most common organism in postoperative endophthalmitis.[1–3,5,6,9,13,22] Gram-negative bacilli were the most common group isolated in our study. *Pseudomonas* was the most common organism cultured. Our finding is in agreement with previous reports from Indian subcontinent. Sharma et al.[24] have reported a high prevalence of gram-negative bacteria and fungus in postoperative endophthalmitis. Anand et al.[25] and Jambulingam et al.[26] have reported similar spectrum in postoperative endophthalmitis from our center. A similar high prevalence of gram-negative bacteria has been reported in other infections in subjects with diabetes.[27] We believe both varied geographical distribution and predominance of subjects with diabetes in our study are responsible for this microbial spectrum.

In this study, a significant number of culture-negative cases could be effectively managed with intravitreal antibiotic injections with relatively better visual and anatomical outcomes. Because the genomic bacterial load predicts severity and outcomes, it is possible that a lower bacterial load favored better outcomes and corresponding inability to capture the microbe on culture.[28] This seems most probable as 75% culture-positive patients presented within 1 week of surgery, in contrast to 66.7% culture-negative patients, owing to this difference in genomic bacterial load.

Overall, the functional outcomes in endophthalmitis after vitrectomy were poor, 24 (63.2%) had final vision <5/200, these results are in accordance with those of previous studies.[1–7,9,12,13] The outcomes are poorer than that of endophthalmitis following cataract surgery due to the possible underlying retinal pathology. The high prevalence of gram-negative and fungal microorganisms in our study partly explains the poor visual outcome in our series. Park et al.[22] reported only 14.8% patients had vision better than 6/12. Scott et al. reported 7 of the 13 cases had a vision worse than 20/100.

The study has several strengths. First, it is the largest case series of endophthalmitis after vitrectomy reported in literature; Second, being a single-center study, it scores over multicenter studies that are confounded by their inherent heterogeneous study design. Third, it addresses the concerns regarding the fear of increased endophthalmitis rate with MIVS using the largest database of 41,921 MIVS surgeries till date. Fourth, the differences between culture-positive and culture-negative cases have been shown in endophthalmitis after vitrectomy. And finally, the use of PCR, a modern sensitive tool used in our study, to exclude probable noninfectious cases has been appropriately demonstrated.

The inherent retrospective study design and unavailability of data on wound construction and suturing are the limitations of our study. Also, as the subgroups have a smaller sample, the true differences in them may be difficult to comment.

In conclusion, using a large dataset, we showed that MIVS does not increase the risk of endophthalmitis, despite recent concerns. Meticulous surgical asepsis, use of endotamponade, suturing of sclerotomy in doubtful cases and judicious use of antibiotics are crucial to minimize the risk of infection. Surgeons should be aware of the differences in presentation, microbiological profile, and factors responsible for unfavorable outcome. The outcomes are often guarded despite appropriate treatment. The risk of poor outcomes is potentially higher in culture-positive cases, which need to be treated aggressively.

## Supporting information

S1 Table Culture sensitivities of organisms in cases with endophthalmitis. (DOCX)

S1 File Highlights. (DOCX)

S2 File IRB certificate. (PDF)

## Abbreviations

BCVA: best-corrected visual acuity
IOAB: intraocular antibiotics
IOP: intraocular pressure
MIVS: minimally invasive vitreoretinal surgery
PCR: polymerase chain reaction
