# Supplementary material for: Incidence, microbiology, and outcomes of endophthalmitis after 111,876 pars plana vitrectomies at a single, tertiary eye care hospital
Source: PLoS One. 2018 Jan 16;13(1):e0191173. doi: 10.1371/journal.pone.0191173 (PMC5770060; doi:10.1371/journal.pone.0191173)
Supplement: S1 Table — (DOCX) [file pone.0191173.s001.docx]

S1 Table: Culture sensitivities of organisms in cases with endophthalmitis.

|  | **Sensitive** | **Resistant** |
| --- | --- | --- |
| Pseudomonas aeruginosa | Gentamicin, Cefotaxime and Amikacin | Tetracycline, Tobramycin, Ampicillin, Ciprofloxacin |
| Aeromonas hydrophila | Gentamicin, Cefotaxime and Tobramycin | Tetracycline, Amikacin, Ampicillin, Ciprofloxacin, Clotriamoxazole |
| Klebsiella ozaenae | Gentamicin, tetracycline, Amikacin, Ciprofloxacin, Ceftazidime and Tobramycin | Ampicillin, Cefotaxime, Cefazolin and Vancomycin |
| Staphylococcus epidermidis | Tetracycline, Tobramycin, Ampicillin, Gentamicin, Ciprofloxacin, Cefotaxime, and Clindamycin | **-** |
| Acinetobacter calcoaceticus | Gentamicin, Cefotaxime, Ciprofloxacin, Amikacin and Ceftazidime | Ampicillin, Clotriamoxazole, Cefazolin and Vancomycin |
| Alcaligenes faecalis | Cefotaxime, Ciprofloxacin, Clotriamoxazole,and ceftazidime | Gentamicin, Ampicillin, Amikacin, Vancomycin, Ceftazidime and Clindamycin |
| Aeromonas hydrophila | Cefotaxime, Ciprofloxacin, Amikacin,and ceftazidime | Gentamicin, and Vancomycin |
| Pseudomonas stutzeri | Gentamicin, Ceftazadime and Amikacin | Cefotoxime and Vancomycin |
| Pseudomonas aeruginosa | Gentamicin, Amikacin, Ciprofloxacin, Cefotoxime, and Ceftazidime | Vancomycin |
| Corynebacterium | Clindamycin | Cephatoxime, Ceftazidime |
| Pseudomonas aeruginosa | Gentamicin, Amikacin, and Ciprofloxacin, | Ceftazidime and Cefotaxime |
| Enterococcus faecalis | Gentamicin, Cefotaxime, Ciprofloxacin, and Vancomycin | Ceftazidime and Clindamycin |
| Staphylococcus aureus | Gentamicin, Cefotaxime, Ciprofloxacin, Vancomycin, Clindamycin, methicillin and Ceftazidime | - |
| Acinetobacter calcoaceticus | Ciprofloxacin, and Amikacin | Gentamicin, Cefotaxime and Ceftazidime |
| Staphylococcus epidermidis | Gentamicin, Amikacin, Ciprofloxacin, Vancomycin, clindamycin, methicillin, penicillin, and Ceftazidime | - |
| Klebsiella oxytoca | Gentamicin, Amikacin, Ciprofloxacin, Cefotoxime, clindamycin and Ceftazedime | - |
| Acinetobacter calcoaceticus | Gentamicin, Ciprofloxacin, Moxifloxacin, Norfloxacin, Tobramycin, Ofloxacin | Cefazolin, Vancomycin |
