# Supplementary material for: Incidence, microbiology, and outcomes of endophthalmitis after 111,876 pars plana vitrectomies at a single, tertiary eye care hospital
Source: PLoS One. 2018 Jan 16;13(1):e0191173. doi: 10.1371/journal.pone.0191173 (PMC5770060; doi:10.1371/journal.pone.0191173)
Supplement: S1 File — (DOCX) [file pone.0191173.s002.docx]

To describe the incidence of endophthalmitis (20 G and minimally invasive vitreoretinal surgery [MIVS]), risk factors, clinical presentation, causative organisms, and outcomes in patients with endophthalmitis after 111,876 Pars Plana Vitrectomies at a Single, Tertiary Eye Care Hospital. We showed that MIVS does not increase the risk of endophthalmitis as compared to 20G vitrectomy.
