# Supplementary material for: Incidence, microbiology, and outcomes of endophthalmitis after 111,876 pars plana vitrectomies at a single, tertiary eye care hospital
Source: PLoS One. 2018 Jan 16;13(1):e0191173. doi: 10.1371/journal.pone.0191173 (PMC5770060; doi:10.1371/journal.pone.0191173)
Supplement: S2 File — (PDF) [file pone.0191173.s003.pdf]

VISION RESEARCH FOUNDATION

[Regd. Under Act XXI of 1860]

New No.41, Old No.18, College Road, Chennai - 600 006

Dr. LINGAM GOPAL, MS., DNBE., FRCS Ed.
President

Dr. S. BHASKARAN, MD., DM (CARD)
Hony. Secretary

R. S. FALOR, B.Com.,FCA, LLB, DBM
Hony. Treasurer

Dr. K. S. VASAN, PhD
Chief Operation Officer

Dr. RONNIE JACOB GEORGE, D.O., D.N.B., M.S
Director-Research

Dr. Muna Bhende
Senior Consultant,
Shri Bhagwan Mahavir Vitreoretinal Services
Medical Research Foundation
Chennai

Dear Sir,

Sub: Institutional Review Board (Ethics Committee) Exemption for the project "Incidence, Microbiology, and Outcomes of Endophthalmitis after 111,876 Pars Plana Vitrectomies at a Single, Tertiary Eye Care Hospital"

Your proposal titled "Incidence, Microbiology, and Outcomes of Endophthalmitis after 111,876 Pars Plana Vitrectomies at a Single, Tertiary Eye Care Hospital", was reviewed by the institution review board and has been approved.

Yours sincerely,

Dr. Ronnie George
Member
Institutional Review Board (Ethics Committee)
Vision Research Foundation
Chennai.

INSTITUTIONAL REVIEW BOARD
(ETHICS COMMITTEE)
VISION RESEARCH FOUNDATION
No. 41 / 18, College Road,
Chennai - 600 006. INDIA

Phone: 91 - 44 - 2827 1616, 4227 1500 **Fax:** 91 - 44 - 2825 4180 **E - mail:** drrg@snmail.org, nanu@snmail.org
WEB: http:// www.sankaranethralaya.org
